# Classification of the Zoonotic Hepatitis E Virus Genotype 3 Into Distinct Subgenotypes

**DOI:** 10.3389/fmicb.2020.634430

**Published:** 2021-01-28

**Authors:** Florence Nicot, Chloé Dimeglio, Marion Migueres, Nicolas Jeanne, Justine Latour, Florence Abravanel, Noémie Ranger, Agnès Harter, Martine Dubois, Sonia Lameiras, Sylvain Baulande, Sabine Chapuy-Regaud, Nassim Kamar, Sébastien Lhomme, Jacques Izopet

**Affiliations:** ^1^CHU de Toulouse, Hôpital Purpan, Laboratoire de Virologie, Centre National de Référence du Virus de l’Hépatite E, Toulouse, France; ^2^INSERM, U1043, Toulouse, France; ^3^Department of Virology, Université Toulouse III Paul-Sabatier, Toulouse, France; ^4^Institut Curie Genomics of Excellence Platform, Institut Curie Research Center, Paris, France; ^5^CHU de Toulouse, Hôpital Rangueil, Service de Néphrologie, Dialyse et Transplantation d’Organe, Toulouse, France

**Keywords:** HEV-3, subtype, classification, diversity, full-length genome, phylogeny

## Abstract

Hepatitis E virus (HEV) genotype 3 is the most common genotype linked to HEV infections in Europe and America. Three major clades (HEV-3.1, HEV-3.2, and HEV-3.3) have been identified but the overlaps between intra-subtype and inter-subtype p-distances make subtype classification inconsistent. Reference sequences have been proposed to facilitate communication between researchers and new putative subtypes have been identified recently. We have used the full or near full-length HEV-3 genome sequences available in the Genbank database (April 2020; *n* = 503) and distance analyses of clades HEV-3.1 and HEV-3.2 to determine a p-distance cut-off (0.093 nt substitutions/site) in order to define subtypes. This could help to harmonize HEV-3 genotyping, facilitate molecular epidemiology studies and investigations of the biological and clinical differences between HEV-3 subtypes.

## Introduction

The hepatitis E virus (HEV) is a significant human pathogen causing viral hepatitis worldwide. Most of the strains that infect humans belong to two species, *Orthohepevirus A* (8 genotypes; HEV 1–8) and *Orthohepevirus C* (Purdy et al., 2017; Smith and Simmonds, 2018; Sridhar et al., 2018; Primadharsini et al., 2019). The most prevalent genotype in industrialized countries at least in Europe and America is HEV genotype 3 (HEV-3). It is transmitted zoonotically by direct contact with infected animals, eating contaminated food, or via the environment. HEV-3 infection is frequently asymptomatic but it can result in severe acute hepatitis in patients with chronic liver disease and lead to chronic hepatitis and cirrhosis in immunocompromised patients (Kamar et al., 2017). Extra-hepatic manifestations have been also described in patients with acute and chronic hepatitis E (Kamar et al., 2017).

Hepatitis E virus genotype 3 variants have been assigned to one of several subtypes based on analysis of a limited number of complete genome sequences and subgenomic regions (Lu et al., 2006). Despite the increasing number of full-length or near full-length genomes deposited in the NCBI database, it is difficult to provide consistent criteria that identify viruses that are members of the same subtype due to overlaps between the intra-subtype and inter-subtype p-distances commonly used for classification (Smith et al., 2015, 2016). Nevertheless, HEV-3 viruses can be classified into three major clades based on phylogenetic grouping. Clade 3.1 includes HEV-3 subtypes a, b, c, h, i, and j; clade 3.2 includes HEV-3 subtypes e, f, and g, and clade 3.3 contains rabbit strains corresponding to the HEV-3ra subtype (Oliveira-Filho et al., 2013; Ijaz et al., 2014; Vina-Rodriguez et al., 2015). A standard reference set of genome sequences including 17 that are full-length or near full-length HEV-3 genomes was proposed in 2016 using a conservative pragmatic approach (Smith et al., 2016). Subsequently, new potential subtypes have been proposed: 3k (Miura et al., 2017), 3l (De Sabato et al., 2018), 3chi-new (Lhomme et al., 2019), and 3s (Wist et al., 2018; Sahli et al., 2019). The standard reference set of genome sequence was recently updated identifying 3k, 3l, and 3m (previously named 3chi-new) as new subtypes (Smith et al., 2020). Recently, an automated partition of phylogenetic trees constructed from 250 full-length HEV-3 genome sequences has been used to classify more than 99% of the complete genome sequences into subtypes (Nicot et al., 2018).

This study was done to determine a distance cut-off that can be used to assign HEV-3 sequences to a subtype using the new full-length HEV-3 sequences available in NCBI and the recently defined new subtypes.

## Materials and Methods

### Sequencing of HEV Complete Genome Sequence

Stored plasma samples from HEV-infected patients consecutively tested for HEV RNA between 2017 and 2019 in the laboratory of Virology at Toulouse University Hospital, National Reference Center for HEV, with viral load of HEV-3 >100,000 copies/mL were selected for PacBio single molecule real-time sequencing. HEV-RNA extraction and F1 and F2 amplifications were realized for 188 samples as previously described (Nicot et al., 2018). SMRT bell library was constructed by pooling 96 barcoded samples according the manufacturer instructions for SMRTbell Barcoded Adapter Prep kit. Sequencing was performed by using chemistry v3.0 on a PacBio Sequel sequencer available at ICGex, Institut Curie Research Center, Paris, France. Bioinformatics analysis and complete genome reconstruction were realized with an in-house developed pipeline. From demultiplexed.bam files provided by ICGex, CCS were constructed using min passes = 3 and min RQ 0.999 parameters. Reads were mapped to a reference sequence (Minimap 2 2.17) to retain HEV reads and remove chimeric reads. Non-identical reads were subsequently combined using a medoïd-based clustering (cluster-fast from USEARCH 11.0.667) into clusters at 99% genetic identity. A consensus sequence was generated for each cluster. F1 and F2 sequences were assembled with Megamerger (EMBOSS 6.6.0). The consensus sequence with the higher number of reads was used as complete genome sequence, annotated as previously described (Nicot et al., 2018) and submitted to Genbank with accession numbers MW355217–MW355404.

### Nucleotide Sequences and Phylogenetic Analysis

The 188 complete genome sequences obtained by SMRT sequencing and all full or near full-length genome sequences of genotype 3 (*n* = 315) of human or animal origin available in the Genbank database on April 2020 were included (Supplementary Table 1). Duplicate sequences from a single individual and six recombinant sequences (D11092, MG783571, KJ013414, KJ013415, KT633715, and DQ450072) were removed. We also included 29 complete genome sequences of genotypes 1, 2, 4, 5, 6, 7, and 8 (Smith et al., 2020) (accession numbers HEV-1: FJ457024, MH918640; HEV-1a: M73218; HEV-1b: L088816; HEV-1c: X98292; HEV-1d: AY230202; HEV-1e: AY204877; HEV-1f: JF443721; HEV-1g: LC225387; HEV-2a: KX578717; HEV-2b: MH809516; HEV-4: MK410048, AB369688; HEV-4a: AB197673; HEV-4b: DQ279091; HEV-4c: AB074915; HEV-4d: AJ272108; HEV–4e: AY723745; HEV-4f: AB220974; HEV-4g: AB108537; HEV-4h: GU119961; HEV-4i: AB369690; HEV-5a: AB573435; HEV-6: AB856243; HEV-6a: AB602441; HEV-7: KJ496144; HEV-7a: KJ496143; and HEV-8: MH410174; HEV-8a: KX387865). The 532 sequences (503 HEV-3 and 29 HEV non-3 genotype) were aligned with MUSCLE v.3.8.31 and a bootstrapped tree (100 replicates) using the maximum likelihood (ML) method with the general time reversible model (GTR + I + G) was constructed on phyML v3.3. Interactive Tree Of Life (iTOL) v3 was used to visualize the whole large tree.

### Automated Partition of Phylogenetic Tree

The ML phylogenetic tree was partitioned and strain clusters within genotype 3 were identified using a method adapted from Prosperi et al. (2011). Briefly, the topology of the ML tree was analyzed with a depth first search by considering the number of subtrees with a node reliability ≥70% and an associated number of leaves with at least two distinct patients. A subtree was identified as a cluster if the median value of the subtree distance distribution was below a t-percentile threshold of the whole-tree distance distribution. If a node satisfied this condition, the search was stopped at that node, children nodes were ignored, and other sibling nodes were analyzed. The threshold *t* was evaluated over the (5th, 50th) percentile range of the whole tree distance distribution with steps of 1 between the 5th and 15th and 5 between the 15th and 50th percentiles.

### Distance Cut-Off for Identification of HEV Genotype 3 Subtypes

The clusters of sequences identified by automated partition of ML phylogenetic tree were used to analyze the intra- and inter-subtype nucleotide pairwise distances. The distances were estimated on MEGA X using the maximum composite likelihood (MCL) method and a gamma distribution to model evolutionary rate differences among sites (four categories). All distances were analyzed (to determine a cut-off that identified subtypes) by generating boxplots in Matlab R2018B software. Based on the algorithm described in Supplementary Figure 1, a sequence *X*_*i*_ can be considered to be a new subtype if all the intra-subtype and inter-subtype distances (*S*_*i,j*_)_*j* ∈ [1,*N*]_ are above a cut-off α. If at least one distance *d*(*X*_*i*_,*X*_*Vj*_),*j* ∈ [1,*N*] between the new sequence *X*_*i*_ and one of the known sequences *X*_*Vj*_ is below the cut-off α, the sequence *X*_*i*_ is assigned to the subtype containing the sequence with the shorter distance.

### Statistical Analysis

Continuous variables were tested with Student’s *t-*test on STATA 14.0 software. *p*-Values of <0.05 were considered to be significant.

## Results

### Identification of Clusters

**FIGURE 1:**
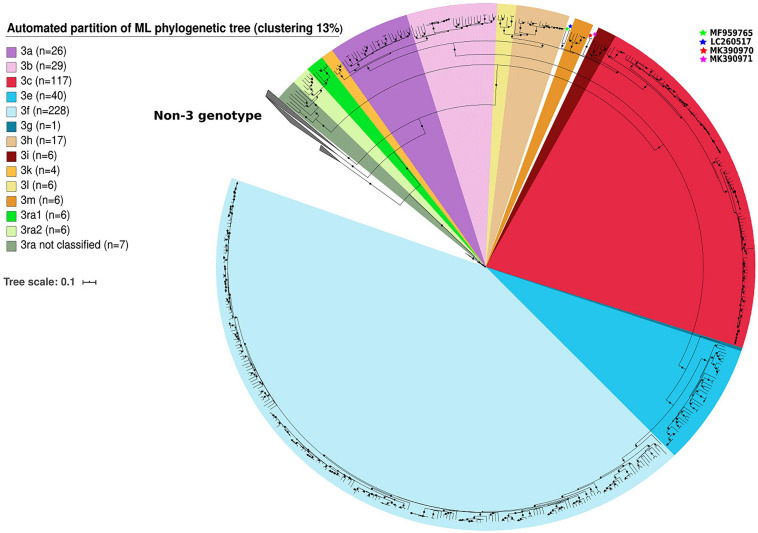
Automated partition of genotype 3 (*n* = 503) clusters within full-length or near full-length genomes. HEV-3 sequences not assigned to a cluster in clades HEV-3.1 are indicated with stars.

We identified clusters among genotype 3 sequences using automated partition of a ML phylogenetic tree. Clusters of known subtypes were identified with a threshold of 13% (median distance <0.126 nt substitutions/site). This threshold assigns 99.1% of the 483 sequences belonging to clade 3.1 and 3.2 to one of the following known subtypes: 3aj (*n* = 26), 3b (*n* = 29), 3c (*n* = 117), 3h (*n* = 17), 3k (*n* = 4), 3i (*n* = 6), 3l (*n* = 6), 3m (*n* = 6) for clade 3.1 and 3e (*n* = 40), 3f (*n* = 228), 3g (*n* = 1) for clade 3.2 (Figure 1). The complete genome of HEV-3j was included in the HEV-3a subtype. The sequences proposed as HEV-3s were classified as HEV-3h subtypes. Only four sequences belonging to the group HEV-3chi (MK390370, MK390371, LC260517, and MF959765) were not classified (Figure 1). The 13% threshold also defined two clusters within clade 3.3 (HEV-3ra) composed of six (HEV-3ra1) and six sequences (HEV-3ra2) while seven sequences were outside the HEV-3ra1 and HEV-3ra2 clusters (Figure 1).

### Determination of a Distance Cut-Off for Subtype Discrimination

**FIGURE 2:**
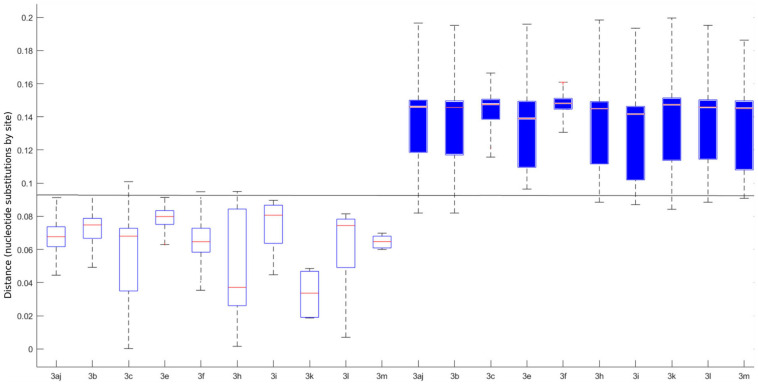
Individual intra (white boxplots) and inter (blue boxplots) p-distances for each determined subtype. The horizontal line represents the 0.093 cut-off. The central mark on each box indicates the median, and the bottom and top edges of the box indicate the 25th and 75th percentiles, respectively. The whiskers extend to the most extreme data points considered the minimum and the maximum of the distribution.

The pairwise distances were estimated on MEGA X using the MCL method. Means for intra-subtype and inter-subtype distances, standard deviation, 95% confidence interval (CI) and 99% CI for each subtype are shown in Table 1. The mean intra-subtype distances obtained for HEV-3ra strains from clade 3.3 (0.116 ± 0.002 nt substitutions/site) was significantly greater than each of the mean intra-subtype distances obtained for the other HEV-3 subtypes (*p* < 0.01 for each subtype). Therefore, the HEV-3ra sequences are too heterogeneous to be used to determine the distance cut-off discriminating between subtypes. Analysis of sequences from clades 3.1 and 3.2 indicated that the overall mean intra-subtype distances was 0.064 (95% CI: 0.03–0.09) whereas the overall mean inter-subtype distances was 0.142 (95% CI: 0.106–0.181). The 95% CI upper limit of intra-subtype distance for each subtype was lower than the 95% CI lower limit of the inter-subtype distance (Table 1). Similarly, the 99% CI upper limit of intra-subtype distance was lower than the 99% CI lower limit of inter-subtype distance, except for subtypes 3aj and 3b. Analysis of the intra- and inter-subtype distances for each subtype indicated that 0.093 can be used as a distance cut-off for assigning a sequence to a subtype (Figure 2). We therefore designed an algorithm based on this cut-off distance that would assign sequences to a subtype (Supplementary Figure 1) and used it to assign 99.1% of the HEV-3 sequences to a defined subtype (3aj, 3b, 3c, 3e, 3f, 3h, 3i, 3k, 3l, 3m). Only four sequences were not assigned (MK390370, MK390371, LC260517, and MF959765), in agreement with the data from automated partition of the ML phylogenetic tree. The distance between sequences MK390370 and MK390371, 0.006 nt substitutions/site, assigned these sequences to the same cluster. LC260517 and MF959765 were isolated sequences (minimum intersubtype-distance: 0.099 nt substitutions/site for both sequences).

**TABLE 1:** Nucleotide p-distances calculated for each subtype (intra and inter-subtype distances).

Subtype	*N*	Distances	Observations	p-distances			
				Mean	SD	95% CI	99% CI
3aj	26	Intra	325	0.067	<0.001	0.066−0.069	0.01−0.096
		Inter	11,804	0.134	<0.001	0.134−0.135	0.089−0.157
3b	29	Intra	406	0.068	<0.001	0.066−0.07	0.003−0.089
		Inter	13,079	0.134	<0.001	0.134−0.135	0.089−0.157
3c	117	Intra	6786	0.055	<0.001	0.055−0.056	0.004−0.085
		Inter	42,471	0.14	<0.001	0.139−0.14	0.1−0.157
3e	40	Intra	780	0.076	<0.001	0.075−0.077	0.002−0.091
		Inter	17,600	0.129	<0.001	0.128−0.129	0.101−0.157
3f	228	Intra	25,878	0.065	<0.001	0.065−0.065	0.019−0.099
		Inter	57,456	0.142	<0.001	0.142−0.142	0.103−0.157
3h	17	Intra	136	0.05	0.003	0.045−0.055	0.002−0.095
		Inter	7871	0.132	<0.001	0.132−0.133	0.098−0.157
3i	6	Intra	15	0.073	0.005	0.062−0.084	0.025−0.09
		Inter	2844	0.127	<0.001	0.126−0.128	0.094−0.152
3k	4	Intra	6	0.033	0.006	0.019−0.048	0.019−0.048
		Inter	1914	0.132	<0.001	0.131−0.133	0.088−0.156
3l	6	Intra	15	0.064	0.006	0.051−0.077	0.007−0.081
		Inter	2844	0.134	<0.001	0.134−0.134	0.093−0.157
3m	6	Intra	15	0.054	0.006	0.042−0.069	0.004−0.069
		Inter	2844	0.131	<0.001	0.13−0.132	0.1−0.156
3ra	17	Intra	171	0.116	0.002	0.111−0.121	0.026−0.161
		Inter	8626	0.181	<0.001	0.181−0.181	0.166−0.193

*p-distances were calculated with the maximum composite likelihood method (MCL) on MEGA X.*

### Classification of HEV-3 Sequences

We used 11 subtypes to classifying sequences in clades 3.1 (3aj, 3b, 3c, 3h, 3i, 3k, 3l, 3m) and 3.2 (3e, 3f, 3g), based on the criteria proposed by Smith et al. (2020) for subtype assignment and the results of the automated partition and distance cut-off methods (Table 2). Each complete genome sequence used in our study was assigned to a subtype (Supplementary Table 1). New complete genome sequences can be assigned to an existing subtype provided there is at least one distance less than 0.093. Otherwise, the new sequence may be a new subtype, which then needs to be confirmed (Smith et al., 2016). Complete genome sequences of subtype 3a, 3b, and 3ra were detected worldwide (Asia, Europe, and America), subtypes 3c, 3e, 3f, and 3h were detected in Asia and Europe, subtypes 3i, 3l, and 3m were detected only in Europe and subtype 3k only in Japan (Table 2 and Supplementary Table 1).

**TABLE 2:** HEV-3 subtype reference sequences based on full or near full-length genomes.

HEV3 subtype according to Smith et al. (2020)	Genbank accession number	Strain	Classification based on clustering or distance analyses	Geographical origin	Comment
3a	AF082843	Meng	3aj	Canada, China, France, Japan, Korea, Mexico, Singapore, Thailand, United Kingdom, United States	
3b	AP003430	JRA1	3b	Canada, China, France, Japan	
3c	FJ705359	wbGER27	3c	France, Germany, Netherlands, Sweden, Thailand, United Kingdom	
3e	AB248521	swJ8-5	3e	Germany, Japan, France, Hungary, Italy, United Kingdom	
3f	AB369687	E116-YKH98C	3f	Denmark, France, Germany, Japan, Singapore, Spain, Sweden, Thailand, United Kingdom	
3g	AF455784	Osh	3g	Kyrgyzstan	Only one complete genome sequence for this subtype
3h	JQ013794	TR19	3h	France, Mongolia, Switzerland	
3i	FJ998008	BB02	3i	France, Germany, Sweden	
3j	AY115488	Arkell	–	Canada	Isolated from pooled stools (Pei and Yoo, 2002), classified 3a
3k	AB369689	E088-STM04C	3k	Japan	
3l	JQ953664	FR-SHEV3c-like	3l	France, Italy	
3m	KU513561	IC2011	3m	France, Spain	
3	AB290313	swMN06-C1056	3f	Mongolia	
3	MF959765	WB/HEV/NA21ITA15	3	Italy	
3	LC260517	swHE1606845	3	Japan	
3	MK390971	17RS1920	3	Italy	
3	MF959764	WB/HEV/NA17ITA15	3i	Italy	
3	KP294371	MWP_2010	3i	Germany	
3ra	FJ906895	GDC9	3ra	China, France, Germany, Japan, United States	

## Discussion

Hepatitis E virus genotype 3 viruses display considerable diversity and have been classified into subtypes with no clear criteria based on distance and phylogenetic methods for demarcation. The set of reference sequences proposed by Smith et al. (2016) has enabled common subtypes to be assigned, but more than 10% of HEV-3 strains were not classified (Nicot et al., 2018). Automated partition of a ML phylogenetic tree using 503 HEV-3 sequences and distance analysis confirmed the classification of 250 HEV-3 sequences (Nicot et al., 2018) and supported the existence of several new post-2016 subtypes included in update classification (Smith et al., 2020). Subtype assignment using our new analysis method is automated and allow the classification of sequences not classified by Smith et al. (2020). It is important to classify a majority of sequences within a subtype and to have an objective method of classification.

The putative subtypes 3k (Miura et al., 2017), 3l (De Sabato et al., 2018), 3m (Nicot et al., 2018; Lhomme et al., 2019), and 3s (Wist et al., 2018; Sahli et al., 2019) have been described. Evidence for subtypes 3k, 3l, and 3m was provided by the automated partition of phylogenetic trees and the distance cut-off of 0.093. HEV-3k has been found in humans and pigs in Japan (Miura et al., 2017) while the HEV-3l subtype was first described in pigs in Northern Italy (De Sabato et al., 2018). Our analyses indicate that HEV-3l subtype also occurs in humans in France (sequences MF444121, MF444131, and HESQL113). The first strain of subtype 3m was detected in a Spanish patient in 2011 (Munoz-Chimeno et al., 2016) and has since been detected in France, Belgium, Netherlands and the United Kingdom (Ijaz et al., 2014; Nicot et al., 2018; Lhomme et al., 2019). A recent study showed that this subtype circulates in wild boar in Spain and also in human in Sweden, suggesting that it is transmitted via consumption of contaminated meat or water or direct contact with wild boar (Wang et al., 2019). In contrast, all the putative subtype 3s sequences were assigned to subtype 3h by the automated partition of ML phylogenetic tree, and distance analysis indicated that 3s sequences should be assigned to subtype 3h, all with distances below the cut-off of 0.093. In addition, these strains, which have been found in both humans and animals in Switzerland, form a cluster that is transmitted by the consumption of locally produced pork meat (Sahli et al., 2019). They cannot be assigned to a new subtype because they are epidemiologically related (Smith et al., 2016). Our analyses also indicate that HEV-3i, described in boar in Germany (Adlhoch et al., 2009) or human in Sweden (Norder et al., 2018), occurs in human in France (HESQL053 and HESQL059).

The sequence AY115488 classified 3j with Smith criteria (Smith et al., 2020) was obtained from the feces of pigs housed in Canada (Pei and Yoo, 2002). This sequence was classified among subtype 3a with our analysis. Indeed, the minimum intra-subtype distance observed between AY115488 and AB089824 (0.083 nucleotide subtitutions/site) in subtype 3aj is much lower than the cut-off value of 0.093 for assigning a sequence to a different subtype. The four sequences (MK390370, MK390371, LC260517, and MF95765) were not classified according Smith et al. (2020) criteria and our analysis. They could be consider as three potential new subtypes, considering MK390370 and MK390371 are assigned to the same cluster. However, in the absence of at least three complete genome sequences epidemiologically unrelated (Smith et al., 2016), these new subtypes could not be confirmed.

Hepatitis E virus genotype 3 is found worldwide and is the predominant genotype in Europe and America. The majority of Asian and North American strains of HEV-3 belong to subtypes 3a and 3b (Zehender et al., 2014). Subtype 3b is indigenous to Japan, although 3b strains have occasionally been identified in Europe (Legrand-Abravanel et al., 2009; Vina-Rodriguez et al., 2015). Subtypes 3k strains have been described only in Asia (Miura et al., 2017). The majority of European strains belong to subtypes 3c, 3f, and 3e. Changes in the distribution of variants within genotypes have been highlighted. A switch from clade 3.2 (mainly 3f and 3e) to clade 3.1 (mainly 3c) infections was observed in France and the United Kingdom after 2010 (Nicot et al., 2018; Oeser et al., 2019) and a similar switch occurred more recently in Belgium; the subtype 3f strains that were predominant before 2015 were replaced by subtype 3c strains after 2015 (Suin et al., 2019). The reason for these changes in subtype distribution is uncertain, it could reflect the distributions of HEV-3 subtypes in the pig reservoirs of different countries. Both locally produced and imported pigs or pork meat could be involved. Phylogenetic and coalescence analyses based on full-length sequences of HEV-3 from acute hepatitis patients, domestic pigs and wild boars provide evidence that HEV-3e strains were introduced from Europe into Japan through importation of pigs in the 1960s (Nakano et al., 2013). Transmission of HEV-3e strains from pigs to wild boars has been also suggested in Japan (Nakano et al., 2013). HEV-3f subtypes were recently detected in humans, domestic pigs and wild mammals in Japan, but indigenous Japanese HEV-3 strains belong to subtypes 3a, 3b, and 3e (Nakano et al., 2018). These new HEV-3f strains may have entered Japan from Europe in this way because the proportion of pork meat imported from Europe has increased in the past decade, leading to cases of hepatitis due to eating pork meat. The changes in HEV-3 subtype distribution are probably the result of changes in the origin of pork meat.

The clinical significance of infection with different HEV-3 subtypes has been discussed. Most studies have shown that asymptomatic blood donors and patients with symptomatic hepatitis E had similar genotype distributions and neither the severity of symptoms nor liver enzyme activities were significantly associated with clades 3.1 or 3.2 (Smith et al., 2015; Lhomme et al., 2019). However, two recent studies from Belgium and France found that the risk of HEV-3-infected patients being hospitalized varied with the subtype (Subissi et al., 2019; Abravanel et al., 2020). Patients infected with subtype 3c were at lower risk of hospitalization than those infected with subtypes 3f or 3e (Subissi et al., 2019; Abravanel et al., 2020). Larger studies are now needed to clarify the influence of host factors and virus diversity on HEV-3 pathogenesis.

A limitation of the present study is the relatively limited number of full length HEV-3 genome sequences available worldwide and the way diversity varies within HEV-3 subtypes. Despite very good groupings of sequences, there are outliers for most subtypes indicating that subtype assignment can be ambiguous. In addition, HEV-3ra strains are particularly heterogeneous. Nevertheless, all HEV-3ra sequences have a common signature, a 93-nucleotide insertion within the macrodomain of the HEV genome (Izopet et al., 2012).

Our findings suggest that the strains in clades 3.1 and 3.2 can be assigned to 1 of 11 subtypes, each represented by a full length or near full-length reference sequence. We have proposed a cut-off value for assigning subtypes. Update of the reference sequences (Smith et al., 2020) could help harmonize HEV-3 classification, which would be useful for comparing strains circulating in humans and the animal reservoir, for tracing the source of an individual infection and for investigating the pathogenicity of HEV-3 subtypes.

## Data Availability Statement

The sequences have been submitted in Genbank database. The accession numbers corresponding the sequences data are MW355217–MW355404.

## Author Contributions

FN and JI designed the project. FN, CD, and JI analyzed the results and wrote the manuscript. NJ, JL, and FN performed the bio-informatics analyses. CD performed the statistics analysis. NK provided the plasma samples. FA, SLh, SC-R, NR, AH, and MD carried out the experiments. SLa and SB performed Sequel sequencing. All authors contributed to the article and approved the submitted version.

## Conflict of Interest

The authors declare that the research was conducted in the absence of any commercial or financial relationships that could be construed as a potential conflict of interest.
