# Supplementary material for: Classification of the Zoonotic Hepatitis E Virus Genotype 3 Into Distinct Subgenotypes
Source: Front Microbiol. 2021 Jan 28;11:634430. doi: 10.3389/fmicb.2020.634430 (PMC7875884; doi:10.3389/fmicb.2020.634430)
Supplement: Supplementary file 1 [file Table_1.DOCX]

**Supplementary Table 1: Subtype of all full or near full-length genomes.**

| **GENBANK ACCESSION NUMBER** | **SUBTYPE ASSIGNMENT** | **HOST** | **COUNTRY** | **COMMENTARY** |
| --- | --- | --- | --- | --- |
| HESQL170 | 3a | human | France |  |
| HESQL016 | 3a | human | France |  |
| AB089824 | 3a | human | Japan |  |
| AB074920 | 3a | human | Japan |  |
| AB074918 | 3a | human | Japan |  |
| AB630970 | 3a | human | Japan |  |
| KT447526 | 3a | human | Singapore |  |
| KT447528 | 3a | human | Singapore |  |
| MH450022 | 3a | human | Thailand |  |
| MH504130 | 3a | human | UK |  |
| HQ389543 | 3a | human | UK |  |
| JN564006 | 3a | human | USA |  |
| AF060669 | 3a | human | USA |  |
| JN837481 | 3a | human | USA |  |
| AF060668 | 3a | human | USA |  |
| AB591734 | 3a | mongoose | Japan |  |
| AY115488 | 3a | swine | Canada | Reference sequence (Smith et al., 2020), 3j |
| KJ507955 | 3a | swine | Canada |  |
| KT727028 | 3a | swine | China |  |
| KX981911 | 3a | swine | China |  |
| KF303502 | 3a | swine | Germany |  |
| AB481228 | 3a | swine | Japan |  |
| FJ426403 | 3a | swine | Korea |  |
| FJ426404 | 3a | swine | Korea |  |
| MG833836 | 3a | swine | Mexico |  |
| AF082843 | 3a | swine | USA | Reference sequence (Smith et al., 2020) |
| AB189071 | 3b | deer | Japan |  |
| AB222182 | 3b | deer | Japan |  |
| AB222183 | 3b | deer | Japan |  |
| AB222184 | 3b | deer | Japan |  |
| HESQL076 | 3b | human | France |  |
| AB291956 | 3b | human | Japan |  |
| AB291952 | 3b | human | Japan |  |
| AB291953 | 3b | human | Japan |  |
| AB291955 | 3b | human | Japan |  |
| AB291951 | 3b | human | Japan |  |
| AB291954 | 3b | human | Japan |  |
| AB291957 | 3b | human | Japan |  |
| AB291960 | 3b | human | Japan |  |
| AB291962 | 3b | human | Japan |  |
| AB369691 | 3b | human | Japan |  |
| AP003430 | 3b | human | Japan | Reference sequence (Smith et al., 2020) |
| AB291963 | 3b | human | Japan |  |
| AB091394 | 3b | human | Japan |  |
| AB630971 | 3b | human | Japan |  |
| AB301710 | 3b | human | Japan |  |
| LC386855 | 3b | human | japan |  |
| AB236320 | 3b | mongoose | Japan |  |
| KJ507956 | 3b | swine | Canada |  |
| FJ527832 | 3b | swine | China |  |
| AB073912 | 3b | swine | Japan |  |
| AB443623 | 3b | swine | Japan |  |
| AB481229 | 3b | swine | Japan |  |
| LC490579 | 3b | swine | japan |  |
| AB246676 | 3b | unknown | Japan |  |
| FJ705359 | 3c | boar | Germany | Reference sequence (Smith et al., 2020) |
| HESQL207 | 3c | human | France |  |
| KJ701409 | 3c | human | France |  |
| MG783570 | 3c | human | France |  |
| HESQL105 | 3c | human | France |  |
| HESQL015 | 3c | human | France |  |
| MG783569 | 3c | human | France |  |
| MF444106 | 3c | human | France |  |
| MF444128 | 3c | human | France |  |
| MF444065 | 3c | human | France |  |
| HESQL082 | 3c | human | France |  |
| MF444122 | 3c | human | France |  |
| HESQL176 | 3c | human | France |  |
| HESQL081 | 3c | human | France |  |
| HESQL148 | 3c | human | France |  |
| MF444072 | 3c | human | France |  |
| HESQL072 | 3c | human | France |  |
| HESQL006 | 3c | human | France |  |
| HESQL115 | 3c | human | France |  |
| HESQL103 | 3c | human | France |  |
| MF444031 | 3c | human | France |  |
| MF444049 | 3c | human | France |  |
| HESQL004 | 3c | human | France |  |
| HESQL035 | 3c | human | France |  |
| HESQL098 | 3c | human | France |  |
| MF444042 | 3c | human | France |  |
| MF444111 | 3c | human | France |  |
| MF444063 | 3c | human | France |  |
| HESQL049 | 3c | human | France |  |
| HESQL104 | 3c | human | France |  |
| HESQL008 | 3c | human | France |  |
| HESQL085 | 3c | human | France |  |
| MF444071 | 3c | human | France |  |
| MF444044 | 3c | human | France |  |
| HESQL061 | 3c | human | France |  |
| HESQL139 | 3c | human | France |  |
| HESQL160 | 3c | human | France |  |
| HESQL112 | 3c | human | France |  |
| HESQL126 | 3c | human | France |  |
| MF444143 | 3c | human | France |  |
| HESQL036 | 3c | human | France |  |
| HESQL091 | 3c | human | France |  |
| HESQL189 | 3c | human | France |  |
| HESQL110 | 3c | human | France |  |
| HESQL102 | 3c | human | France |  |
| HESQL010 | 3c | human | France |  |
| HESQL021 | 3c | human | France |  |
| HESQL175 | 3c | human | France |  |
| HESQL149 | 3c | human | France |  |
| HESQL209 | 3c | human | France |  |
| HESQL133 | 3c | human | France |  |
| HESQL029 | 3c | human | France |  |
| HESQL041 | 3c | human | France |  |
| HESQL146 | 3c | human | France |  |
| HESQL145 | 3c | human | France |  |
| HESQL003 | 3c | human | France |  |
| HESQL166 | 3c | human | France |  |
| HESQL034 | 3c | human | France |  |
| HESQL022 | 3c | human | France |  |
| HESQL109 | 3c | human | France |  |
| HESQL197 | 3c | human | France |  |
| HESQL031 | 3c | human | France |  |
| HESQL114 | 3c | human | France |  |
| HESQL028 | 3c | human | France |  |
| HESQL195 | 3c | human | France |  |
| HESQL024 | 3c | human | France |  |
| HESQL033 | 3c | human | France |  |
| HESQL204 | 3c | human | France |  |
| HESQL210 | 3c | human | France |  |
| HESQL143 | 3c | human | France |  |
| HESQL153 | 3c | human | France |  |
| HESQL025 | 3c | human | France |  |
| HESQL039 | 3c | human | France |  |
| HESQL044 | 3c | human | France |  |
| HESQL100 | 3c | human | France |  |
| HESQL009 | 3c | human | France |  |
| HESQL129 | 3c | human | France |  |
| HESQL017 | 3c | human | France |  |
| HESQL020 | 3c | human | France |  |
| HESQL121 | 3c | human | France |  |
| HESQL005 | 3c | human | France |  |
| MF444064 | 3c | human | France |  |
| HESQL167 | 3c | human | France |  |
| KU176129 | 3c | human | France |  |
| HESQL023 | 3c | human | France |  |
| HESQL162 | 3c | human | France |  |
| HESQL138 | 3c | human | France |  |
| MF444115 | 3c | human | France |  |
| MF444085 | 3c | human | France |  |
| HESQL135 | 3c | human | France |  |
| MF444114 | 3c | human | France |  |
| MF444043 | 3c | human | France |  |
| HESQL007 | 3c | human | France |  |
| HESQL136 | 3c | human | France |  |
| HESQL054 | 3c | human | France |  |
| KX172133 | 3c | human | Germany |  |
| MK089849 | 3c | human | Germany |  |
| KC618402 | 3c | human | Germany |  |
| MN614140 | 3c | human | Netherlands |  |
| MH377727 | 3c | human | Sweden |  |
| MH377723 | 3c | human | Sweden |  |
| MH377722 | 3c | human | Sweden |  |
| MH450021 | 3c | human | Thailand |  |
| KX462160 | 3c | human | UK |  |
| MH504126 | 3c | human | UK |  |
| MH504132 | 3c | human | UK |  |
| MH504124 | 3c | human | UK |  |
| MH504133 | 3c | human | UK |  |
| MH504128 | 3c | human | UK |  |
| MH504137 | 3c | human | UK |  |
| MH504136 | 3c | human | UK |  |
| MH504131 | 3c | human | UK |  |
| MH504135 | 3c | human | UK |  |
| MH504127 | 3c | human | UK |  |
| MH504138 | 3c | human | UK |  |
| KT159771 | 3c | human | UK |  |
| MH504134 | 3c | human | UK |  |
| FJ998015 | 3e | boar | Germany |  |
| AB780450 | 3e | boar | Japan |  |
| AB780452 | 3e | boar | Japan |  |
| AB780451 | 3e | boar | Japan |  |
| AB780453 | 3e | boar | Japan |  |
| JQ013795 | 3e | human | France |  |
| HESQL123 | 3e | human | France |  |
| MF444141 | 3e | human | France |  |
| MF444109 | 3e | human | France |  |
| KF922359 | 3e | human | France |  |
| MF444086 | 3e | human | France |  |
| MH377724 | 3e | human | Germany |  |
| MK089848 | 3e | human | Germany |  |
| AB248520 | 3e | human | Japan |  |
| AB291958 | 3e | human | Japan |  |
| MH504144 | 3e | human | UK |  |
| MH504149 | 3e | human | UK |  |
| MH504141 | 3e | human | UK |  |
| MH504142 | 3e | human | UK |  |
| MH504143 | 3e | human | UK |  |
| MH504140 | 3e | human | UK |  |
| MH504152 | 3e | human | UK |  |
| MH504139 | 3e | human | UK |  |
| MH504150 | 3e | human | UK |  |
| MH504146 | 3e | human | UK |  |
| MH504145 | 3e | human | UK |  |
| MH504148 | 3e | human | UK |  |
| MH504153 | 3e | human | UK |  |
| JQ026407 | 3e | monkey | Japan |  |
| JQ953665 | 3e | swine | France |  |
| HM055578 | 3e | swine | Hungary |  |
| KP698919 | 3e | swine | Italy |  |
| AB248522 | 3e | swine | Japan |  |
| AB481226 | 3e | swine | Japan |  |
| AB248521 | 3e | swine | Japan | Reference sequence (Smith et al., 2020) |
| MH184581 | 3e | swine | UK |  |
| MH184582 | 3e | swine | UK |  |
| MH184583 | 3e | swine | UK |  |
| MH184579 | 3e | swine | UK |  |
| MH184584 | 3e | swine | UK |  |
| HESQL055 | 3f | human | France |  |
| HESQL147 | 3f | human | France |  |
| MF444080 | 3f | human | France |  |
| HESQL111 | 3f | human | France |  |
| HESQL013 | 3f | human | France |  |
| MF444091 | 3f | human | France |  |
| MF444053 | 3f | human | France |  |
| HESQL067 | 3f | human | France |  |
| HESQL108 | 3f | human | France |  |
| HESQL211 | 3f | human | France |  |
| HESQL169 | 3f | human | France |  |
| HESQL185 | 3f | human | France |  |
| HESQL043 | 3f | human | France |  |
| HESQL155 | 3f | human | France |  |
| MF444075 | 3f | human | France |  |
| MF444144 | 3f | human | France |  |
| MF444113 | 3f | human | France |  |
| HESQL137 | 3f | human | France |  |
| HESQL001 | 3f | human | France |  |
| HESQL152 | 3f | human | France |  |
| MF444045 | 3f | human | France |  |
| HESQL151 | 3f | human | France |  |
| HESQL161 | 3f | human | France |  |
| MF444087 | 3f | human | France |  |
| MF444134 | 3f | human | France |  |
| HESQL092 | 3f | human | France |  |
| HESQL128 | 3f | human | France |  |
| MF444123 | 3f | human | France |  |
| MF444107 | 3f | human | France |  |
| MF444137 | 3f | human | France |  |
| MF444036 | 3f | human | France |  |
| MF444057 | 3f | human | France |  |
| HESQL118 | 3f | human | France |  |
| HESQL158 | 3f | human | France |  |
| HESQL107 | 3f | human | France |  |
| HESQL083 | 3f | human | France |  |
| HESQL174 | 3f | human | France |  |
| HESQL120 | 3f | human | France |  |
| HESQL070 | 3f | human | France |  |
| HESQL019 | 3f | human | France |  |
| HESQL095 | 3f | human | France |  |
| MF444133 | 3f | human | France |  |
| MF444095 | 3f | human | France |  |
| MF444108 | 3f | human | France |  |
| MF444084 | 3f | human | France |  |
| MF444060 | 3f | human | France |  |
| MF444040 | 3f | human | France |  |
| HESQL156 | 3f | human | France |  |
| HESQL068 | 3f | human | France |  |
| HESQL060 | 3f | human | France |  |
| HESQL116 | 3f | human | France |  |
| HESQL168 | 3f | human | France |  |
| HESQL086 | 3f | human | France |  |
| HESQL065 | 3f | human | France |  |
| HESQL056 | 3f | human | France |  |
| HESQL087 | 3f | human | France |  |
| MF444054 | 3f | human | France |  |
| MF444061 | 3f | human | France |  |
| HESQL205 | 3f | human | France |  |
| MF444097 | 3f | human | France |  |
| MF444048 | 3f | human | France |  |
| MF444096 | 3f | human | France |  |
| MF444047 | 3f | human | France |  |
| MF444132 | 3f | human | France |  |
| MF444038 | 3f | human | France |  |
| HESQL165 | 3f | human | France |  |
| HESQL084 | 3f | human | France |  |
| MF444130 | 3f | human | France |  |
| MF444066 | 3f | human | France |  |
| MF444050 | 3f | human | France |  |
| MF444093 | 3f | human | France |  |
| MF444139 | 3f | human | France |  |
| MF444055 | 3f | human | France |  |
| MF444124 | 3f | human | France |  |
| MF444073 | 3f | human | France |  |
| MF444142 | 3f | human | France |  |
| MF444083 | 3f | human | France |  |
| HESQL051 | 3f | human | France |  |
| HESQL046 | 3f | human | France |  |
| MF444126 | 3f | human | France |  |
| MF444051 | 3f | human | France |  |
| HESQL073 | 3f | human | France |  |
| HESQL040 | 3f | human | France |  |
| MF444098 | 3f | human | France |  |
| MF444088 | 3f | human | France |  |
| HESQL097 | 3f | human | France |  |
| MF444081 | 3f | human | France |  |
| HESQL066 | 3f | human | France |  |
| EU495148 | 3f | human | France |  |
| MF444058 | 3f | human | France |  |
| MF444105 | 3f | human | France |  |
| MF444082 | 3f | human | France |  |
| HESQL063 | 3f | human | France |  |
| MF444094 | 3f | human | France |  |
| HESQL184 | 3f | human | France |  |
| HESQL188 | 3f | human | France |  |
| HESQL124 | 3f | human | France |  |
| MF444035 | 3f | human | France |  |
| HESQL057 | 3f | human | France |  |
| MF444039 | 3f | human | France |  |
| MF444112 | 3f | human | France |  |
| MF444100 | 3f | human | France |  |
| MF444103 | 3f | human | France |  |
| MF444138 | 3f | human | France |  |
| HESQL002 | 3f | human | France |  |
| HESQL048 | 3f | human | France |  |
| HESQL180 | 3f | human | France |  |
| HESQL071 | 3f | human | France |  |
| HESQL131 | 3f | human | France |  |
| HESQL018 | 3f | human | France |  |
| HESQL011 | 3f | human | France |  |
| HESQL069 | 3f | human | France |  |
| HESQL117 | 3f | human | France |  |
| MF444140 | 3f | human | France |  |
| HESQL094 | 3f | human | France |  |
| HESQL142 | 3f | human | France |  |
| HESQL206 | 3f | human | France |  |
| HESQL212 | 3f | human | France |  |
| HESQL208 | 3f | human | France |  |
| HESQL062 | 3f | human | France |  |
| HESQL177 | 3f | human | France |  |
| HESQL186 | 3f | human | France |  |
| HESQL026 | 3f | human | France |  |
| HESQL090 | 3f | human | France |  |
| MF444069 | 3f | human | France |  |
| HESQL079 | 3f | human | France |  |
| HESQL096 | 3f | human | France |  |
| MF444090 | 3f | human | France |  |
| HESQL101 | 3f | human | France |  |
| MF444125 | 3f | human | France |  |
| MF444070 | 3f | human | France |  |
| HESQL045 | 3f | human | France |  |
| MN401237 | 3f | human | France |  |
| HESQL042 | 3f | human | France |  |
| MF444076 | 3f | human | France |  |
| HESQL194 | 3f | human | France |  |
| MF444092 | 3f | human | France |  |
| MF444129 | 3f | human | France |  |
| MF444127 | 3f | human | France |  |
| MF444034 | 3f | human | France |  |
| MF444027 | 3f | human | France |  |
| MF444117 | 3f | human | France |  |
| MF444041 | 3f | human | France |  |
| MF444078 | 3f | human | France |  |
| HESQL202 | 3f | human | France |  |
| HESQL027 | 3f | human | France |  |
| MF444119 | 3f | human | France |  |
| MF444059 | 3f | human | France |  |
| KC166967 | 3f | human | France |  |
| JN906974 | 3f | human | France |  |
| MF444102 | 3f | human | France |  |
| MF444033 | 3f | human | France |  |
| MF444052 | 3f | human | France |  |
| MF444135 | 3f | human | France |  |
| HESQL014 | 3f | human | France |  |
| HESQL181 | 3f | human | France |  |
| HESQL130 | 3f | human | France |  |
| HESQL125 | 3f | human | France |  |
| MF444067 | 3f | human | France |  |
| HESQL134 | 3f | human | France |  |
| HESQL191 | 3f | human | France |  |
| HESQL078 | 3f | human | France |  |
| HESQL132 | 3f | human | France |  |
| HESQL172 | 3f | human | France |  |
| HESQL058 | 3f | human | France |  |
| MF444032 | 3f | human | France |  |
| HESQL064 | 3f | human | France |  |
| MF444029 | 3f | human | France |  |
| MF444062 | 3f | human | France |  |
| MF444104 | 3f | human | France |  |
| HESQL150 | 3f | human | France |  |
| HESQL075 | 3f | human | France |  |
| HESQL190 | 3f | human | France |  |
| HESQL178 | 3f | human | France |  |
| MF444101 | 3f | human | France |  |
| MF444046 | 3f | human | France |  |
| HESQL080 | 3f | human | France |  |
| MF444079 | 3f | human | France |  |
| HESQL047 | 3f | human | France |  |
| KT591532 | 3f | human | France |  |
| MF444028 | 3f | human | France |  |
| HESQL144 | 3f | human | France |  |
| HESQL164 | 3f | human | France |  |
| HESQL012 | 3f | human | France |  |
| MF444118 | 3f | human | France |  |
| MF444116 | 3f | human | France |  |
| HESQL119 | 3f | human | France |  |
| HESQL038 | 3f | human | France |  |
| HESQL032 | 3f | human | France |  |
| HESQL154 | 3f | human | France |  |
| HESQL159 | 3f | human | France |  |
| HESQL077 | 3f | human | France |  |
| HESQL157 | 3f | human | France |  |
| MK089847 | 3f | human | Germany |  |
| KU980235 | 3f | human | Germany |  |
| FJ956757 | 3f | human | Germany |  |
| AB850879 | 3f | human | Japan |  |
| LC055972 | 3f | human | Japan |  |
| AB291961 | 3f | human | Japan |  |
| AB369687 | 3f | human | Japan | Reference sequence (Smith et al., 2020) |
| KT447527 | 3f | human | Singapore |  |
| MH377725 | 3f | human | Sweden |  |
| MH377726 | 3f | human | Sweden |  |
| MH450020 | 3f | human | Thailand |  |
| KY232312 | 3f | human | Thailand |  |
| FJ653660 | 3f | human | Thailand |  |
| MH504147 | 3f | human | UK |  |
| MH504151 | 3f | human | UK |  |
| KU747141 | 3f | swine | Denmark |  |
| KU747142 | 3f | swine | Denmark |  |
| JQ953666 | 3f | swine | France |  |
| AB290313 | 3f | swine | Mongolia | Reference sequence (Smith et al., 2020), subtype not assigned |
| EU723512 | 3f | swine | Spain |  |
| EU723513 | 3f | swine | Spain |  |
| EU723516 | 3f | swine | Spain |  |
| EU723514 | 3f | swine | Spain |  |
| EU723515 | 3f | swine | Spain |  |
| EU360977 | 3f | swine | Sweden |  |
| MH450030 | 3f | swine | Thailand |  |
| MH450031 | 3f | swine | Thailand |  |
| MH450023 | 3f | swine | Thailand |  |
| MH450027 | 3f | swine | Thailand |  |
| MH450028 | 3f | swine | Thailand |  |
| MH450029 | 3f | swine | Thailand |  |
| MH450025 | 3f | swine | Thailand |  |
| MH450026 | 3f | swine | Thailand |  |
| MH450024 | 3f | swine | Thailand |  |
| EU375463 | 3f | swine | Thailand |  |
| AF455784 | 3g | swine | Kyrgyzstan | Reference sequence (Smith et al., 2020) |
| MF444056 | 3h | human | France |  |
| HESQL074 | 3h | human | France |  |
| MF444120 | 3h | human | France |  |
| JQ013794 | 3h | human | France | Reference sequence (Smith et al., 2020) |
| HESQL122 | 3h | human | France |  |
| HESQL187 | 3h | human | France |  |
| MF444145 | 3h | human | France |  |
| KU176132 | 3h | human | France |  |
| MF444110 | 3h | human | France |  |
| MF444136 | 3h | human | France |  |
| MF444077 | 3h | human | France |  |
| MF444037 | 3h | human | France |  |
| KU176131 | 3h | human | France |  |
| KY780957 | 3h | human | Switzerland |  |
| MF346772 | 3h | human | Switzerland |  |
| AB290312 | 3h | swine | Mongolia |  |
| MG573193 | 3h | swine | Switzerland |  |
| KP294371 | 3i | boar | Germany | Reference sequence (Smith et al., 2020), subtype not assigned |
| FJ998008 | 3i | boar | Germany | Reference sequence (Smith et al., 2020) |
| MF959764 | 3i | boar | Italy | Reference sequence (Smith et al., 2020), subtype not assigned |
| HESQL053 | 3i | human | France |  |
| HESQL059 | 3i | human | France |  |
| MH377721 | 3i | human | Sweden |  |
| AB740232 | 3k | boar | Japan |  |
| AB369689 | 3k | human | Japan | Reference sequence (Smith et al., 2020) |
| LC131066 | 3k | human | Japan |  |
| LC176492 | 3k | human | Japan |  |
| MF444121 | 3l | human | France |  |
| HESQL113 | 3l | human | France |  |
| MF444131 | 3l | human | France |  |
| JQ953664 | 3l | swine | France | Reference sequence (Smith et al., 2020) |
| KY766999 | 3l | swine | Italy |  |
| MG674164 | 3l | swine | Italy |  |
| HESQL093 | 3m | human | France |  |
| MF444030 | 3m | human | France |  |
| HESQL050 | 3m | human | France |  |
| KU176130 | 3m | human | France |  |
| MF444089 | 3m | human | France |  |
| KU513561 | 3m | human | Spain | Reference sequence (Smith et al., 2020) |
| LY647099 | 3ra1 | rabbit | China |  |
| GU937805 | 3ra1 | rabbit | China |  |
| JQ768461 | 3ra1 | rabbit | China |  |
| AB740221 | 3ra1 | rabbit | China |  |
| FJ906896 | 3ra1 | rabbit | China |  |
| LC484431 | 3ra1 | rabbit | Japan |  |
| MF444099 | 3ra2 | human | France |  |
| AB740220 | 3ra2 | rabbit | China |  |
| KX227751 | 3ra2 | rabbit | China |  |
| AB740222 | 3ra2 | rabbit | China |  |
| FJ906895 | 3ra2 | rabbit | China | Reference sequence (Smith et al., 2020) |
| JX565469 | 3ra2 | rabbit | USA |  |
| MG211750 | 3ra not classified | human | France |  |
| MF444074 | 3ra not classified | human | France |  |
| JQ013793 | 3ra not classified | human | France |  |
| JQ013792 | 3ra not classified | rabbit | France |  |
| JQ013791 | 3ra not classified | rabbit | France |  |
| MK050463 | 3ra not classified | rabbit | Germany |  |
| KY436898 | 3ra not classified | rabbit | Germany |  |
| MF959765 | not assigned | boar | Italy | Reference sequence (Smith et al., 2020), subtype not assigned |
| MK390971 | not assigned | boar | Italy | Reference sequence (Smith et al., 2020), subtype not assigned |
| MK390970 | not assigned | boar | Italy |  |
| LC260517 | not assigned | swine | Japon | Reference sequence (Smith et al., 2020), subtype not assigned |
