# Supplementary figures and images for: Classification of the Zoonotic Hepatitis E Virus Genotype 3 Into Distinct Subgenotypes
Source: Front Microbiol. 2021 Jan 28;11:634430. doi: 10.3389/fmicb.2020.634430 (PMC7875884; doi:10.3389/fmicb.2020.634430)

**Supplementary Figure 1: Algorithm used to classify unknown sequences**


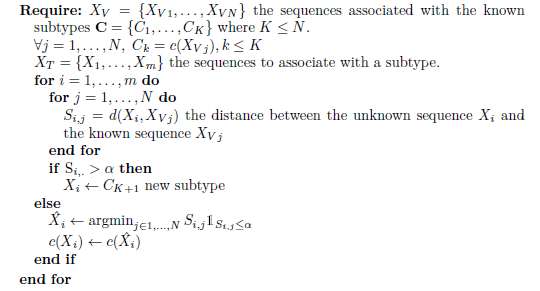

Supplement: Supplementary file 2 [file Table_2.DOCX]
